# Identification of Respiratory Pauses during Swallowing by Unconstrained Measuring Using Millimeter Wave Radar

**DOI:** 10.3390/s24123748

**Published:** 2024-06-09

**Authors:** Toma Kadono, Hiroshi Noguchi

**Affiliations:** Graduate School of Engineering, Osaka Metropolitan University, 1-1 Gakuencho, Nakaku, Osaka 599-8531, Japan

**Keywords:** millimeter wave radar, machine learning, swallowing, respiratory waveform, unconstrained measuring

## Abstract

Breathing temporarily pauses during swallowing, and the occurrence of inspiration before and after these pauses may increase the likelihood of aspiration, a serious health problem in older adults. Therefore, the automatic detection of these pauses without constraints is important. We propose methods for measuring respiratory movements during swallowing using millimeter wave radar to detect these pauses. The experiment involved 20 healthy adult participants. The results showed a correlation of 0.71 with the measurement data obtained from a band-type sensor used as a reference, demonstrating the potential to measure chest movements associated with respiration using a non-contact method. Additionally, temporary respiratory pauses caused by swallowing were confirmed by the measured data. Furthermore, using machine learning, the presence of respiring alone was detected with an accuracy of 88.5%, which is higher than that reported in previous studies. Respiring and temporary respiratory pauses caused by swallowing were also detected, with a macro-averaged F1 score of 66.4%. Although there is room for improvement in temporary pause detection, this study demonstrates the potential for measuring respiratory movements during swallowing using millimeter wave radar and a machine learning method.

## 1. Introduction

Maintaining normal swallowing function is crucial for older adults to sustain their health through proper nutritional intake and a satisfactory eating experience. Swallowing is achieved by the coordinated action of multiple muscles and nerves, and loss of this coordination can lead to swallowing disorders. A typical disease resulting from swallowing disorders is aspiration pneumonia, caused by food entering the airways or lungs, leading to bacterial growth. Pneumonia is a major cause of death in older adults, with aspiration being a significant risk factor [1]. Research targeting pneumonia patients aged 70 and above has shown that approximately 80% of them have swallowing disorders [2]. Additionally, aspiration can cause complications or sequelae of other diseases. For instance, throat muscle paralysis due to a stroke increases the risk of developing swallowing disorders [3,4]. Patients with Parkinson’s, a neurological disorder, often experience concurrent swallowing difficulties [5]. Numerous studies have focused on patients with Parkinson’s [6,7]. Furthermore, swallowing difficulties are not only complications or sequelae but also factors contributing to a decline in the quality of life (QoL) of patients. A decreased ability to swallow can lead to reduced appetite, exacerbating the risk of deterioration in nutritional status and muscle weakness, among other diseases [8]. Early recognition and intervention are crucial for extending healthy life expectancy and improving QoL.

Therefore, diagnosing dysphagia and assessing swallowing ability is necessary. Currently, the videofluoroscopic swallowing evaluation [9], which diagnoses dysphagia from X-ray images inside the throat, and videoendoscopic evaluation of dysphagia [10], which uses endoscopy for examination, are widely used as diagnostic methods for dysphagia. However, these methods are invasive and burdensome for patients and require specialized equipment and measurement environments, making them unsuitable for daily assessments. To overcome these problems, another evaluation method involves assessing the process of beverage swallowing [11]. In caregiving settings, screening tests for assessing swallowing ability such as the repetitive saliva swallowing test (RSST) [12] and the modified water swallowing test (MWST) [13] are used. However, these tests require special actions, such as placing food on the dorsum of the tongue and checking for oral residue. These actions can impose significant burdens on caregivers and older adults. Consequently, there is an increasing demand for new methods that can be easily incorporated into daily routines.

Swallowing is primarily divided into three phases: oral, pharyngeal, and oesophageal [14]. In this study, we focused on the pharyngeal phase, which involves propelling food from the back of the throat into the esophagus, because this phase is directly related to aspiration. During this phase, laryngeal elevation occurs unconsciously to prevent the entry of the bolus into the trachea, and the epiglottis blocks the trachea. A temporary respiratory pause occurs simultaneously due to tracheal occlusion, and measurements using endoscopy and respiratory plethysmography have shown coordination between laryngeal elevation and respiratory pauses by swallowing [15]. In this study, a respiratory pause was defined as the period from the onset of laryngeal elevation to the onset of descent. Additionally, attention has been drawn to the coordination between swallowing and breathing, particularly the pattern of inspiration that occurs immediately before or after swallowing. Disrupted coordination between swallowing and breathing may be associated with an increased risk of aspiration. This association is supported by the fact that patients with swallowing disorders or Parkinson’s disease have a higher frequency of these occurrences than healthy individuals [16,17]. Therefore, measuring respiratory pauses during swallowing is crucial for detecting the risk of aspiration.

Therefore, measuring respiratory movements during swallowing is beneficial for evaluating swallowing ability and could lead to the early detection of a decline in physical function or specific diseases. A non-invasive method for measuring respiratory activity involves the use of a nasal cannula to measure nasal airflow. Research has been conducted on the mechanism of measuring the coordination between swallowing and breathing by combining a nasal cannula with a pressure sensor and surface electromyography (sEMG) for the swallowing measurements [18,19]. However, these methods utilize contact sensors that require wearing of instruments, which may pose concerns regarding inconvenience, difficulty in application, and discomfort during measurement. Considering that dysphagia is commonly observed in older adults, avoiding such issues is essential. Therefore, development of unconstrained methods for respiratory measurements is required.

One method of conducting unconstrained respiratory measurements is to measure breathing during sleep by placing pressure sensors on beds or pillows to detect respiration and movement [20,21]. While this approach could be applicable for measuring breathing during swallowing in this study, it is limited to measurements during sleep, which means that it cannot measure breathing while swallowing during meals. Another method involves the use of cameras to measure the respiratory cycles [22], but privacy concerns exist.

Therefore, another method is required to measure the subtle chest movements caused by breathing. During breathing, the volume of the chest cavity, which constitutes the outer shell of the chest, is comprised of ribs that expand and contract. Respiration occurs through the repetition of this movement, resulting in overall movement of the chest and abdomen. To measure this unconstrained movement, radar was used to measure the distance to the surface of the chest. The radar uses radio waves to perform distance measurements, unlike stereo cameras or infrared sensors. Previous studies have achieved distance measurements using radar to capture body movements and respiratory cycles without contact [23,24]. Since radio waves penetrate clothing, measurements can be performed even when the subject is clothed. Therefore, radar is a promising non-contact and routine measurement method.

However, research measuring respiratory movements during swallowing using radar is lacking. We hypothesized that this radar-based respiratory measurement method could confirm temporary respiratory pauses during swallowing and the coordination between swallowing and breathing. The natural movement of the chest during breathing is less than 10 mm [25], making it challenging to capture this movement accurately. While previous studies have utilized ultra-high frequency (UHF) [23] and microwave frequencies [24] for radar, radar technology utilizing higher-frequency waves, millimeter waves, has become prevalent in recent years. Millimeter waves are a frequency band in high demand, particularly for the advancement of communication technologies such as 5G. Various research efforts are underway to utilize millimeter waves, including their use in meteorological radar systems [26] and for the miniaturization, high efficiency, and cost reduction of antennas [27,28]. We chose to use millimeter waves as a sensor for distance measurements.

We speculate that by using millimeter wave radar, which enables measurements with shorter wavelengths and smaller distances, it may be possible to accurately measure subtle changes in chest movements related to swallowing. Several studies have addressed respiration detection using millimeter waves [29,30,31]. These studies used frequency analysis to leverage the periodic nature of respiration. However, swallowing is a non-periodic phenomenon. Moreover, while Kubo et al. [24] and Shah et al. [29] demonstrated the detection of breath-holding for several seconds, the cessation of respiration due to swallowing is much shorter, making its detection a more challenging task. Therefore, a novel approach for swallowing detection is required.

**Figure 1:**
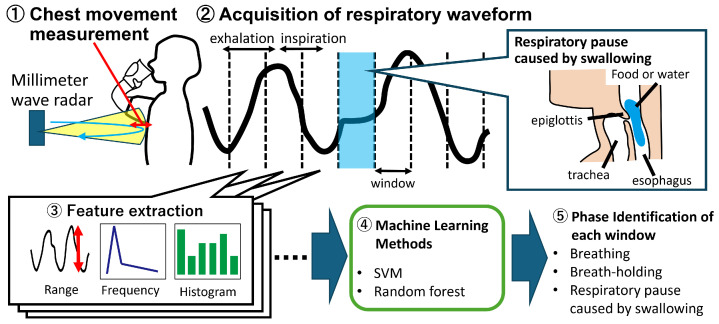
Overall procedure of the proposed method for identifying temporary respiratory pauses during swallowing.

Furthermore, owing to individual differences in factors such as respiratory cycles, the duration of temporary respiratory pauses caused by swallowing, and variations in noise levels among the data. Machine learning was used as the detection method to account for these variations. This study aimed to develop a respiratory measurement method based on chest movements using millimeter wave radar and to detect the presence of breathing and temporary respiratory pauses caused by swallowing using machine learning techniques. Figure 1 illustrates the overall procedure for measurement and identification.

## 2. Used Millimeter Wave Radar

In this study, XM112 (Acconeer, Sweden) [32], which operates at 60 GHz millimeter waves, was used. The frequency band of the radio waves ranged from 57 GHz to 64 GHz, with a central frequency of 60.5 GHz. This frequency is allocated for millimeter wave radar. Therefore, they are less susceptible to interference from other radio waves when used indoors. The sensor includes a chip for transmitting and receiving millimeter waves, a microcontroller unit for processing the transmitted and received data, and a breakout board for transmitting the data to a PC via USB serial communication. By using radar which allows for non-contact measurement instead of sEMG or pressure sensors, measurements can be achieved without interfering with daily life. The size of this sensor is 43×35 mm^2^. This is smaller than that of the automotive millimeter wave radar used in related studies [29,30,31], making it easier to instal robots for monitoring the elderly. The maximum output power of the sensor is 10 dBm, equivalent to 10 mW. This power level is extremely low compared to that of devices using conventional radio waves, making it safe without adverse effects on the human body. The maximum measurement distance is 2 m, which is sufficient for this study. The range of this radar, the half-power beam width (the angle at which the power value is halved relative to the forward direction), is $45^{\circ }$ in the vertical direction and $70^{\circ }$ in the horizontal direction. Therefore, this millimeter wave radar can be used as a small sensor to measure the movement of the entire chest.

Distance sensors commonly employ lasers or infrared radiation and are used in systems for detecting people and objects. These sensors typically use the time of flight (ToF) principle. The ToF method involves transmitting a signal and measuring the time required for the signal output to be reflected. The distance is calculated based on this time difference. However, using this method, only a time change of approximately $7\times 10^{-12}$ s occurs for a distance change of 1 mm. Considering its accuracy and resolution, it is difficult to measure chest movements of less than 10 mm with small sensors that can be introduced into a home using this method. However, the radar can achieve distance measurements with higher accuracy by detecting the phases of reflected waves. The relation between the phase changes Δϕ is expressed by Equation (1), where ΔR represents the variation in distance, *f* is the frequency of the radio wave, and *c* is the speed of light.

(1) $$\Delta \phi =\frac{4\pi \Delta Rf}{c}$$

At 60 GHz, a movement of 1 mm is extracted with a phase change of approximately $144^{\circ }$. This indicates that slight changes in the distance can be detected using large phase changes. Furthermore, Δϕ and *f* are proportional. Thus, the use of a high-frequency radar indicates the ability to detect minute distance changes. For example, compared with the microwave (24 GHz) radar used by Kubo et al. [24], this radar has more than twice the sensitivity. Furthermore, in this study, radio waves were radiated in a radial pattern and directed to the entire chest to measure the chest movements caused by respiring and swallowing.

This sensor employs a pulse-coherent radar method, which involves transmitting pulse waves with phase information at each sampling interval and measuring the distance from the reflected waves to the object. In this process, the use of a 24 MHz clock with a quartz oscillator allows for precise control of the transmission intervals and phases. By transmitting pulse waves with the same phase at each interval, accurately measuring the position based on the phase variation of the received pulses was possible. According to the datasheet of this radar, the precision of phase control was evaluated in experiments conducted at a temperature of 25 °C and a distance of 0.35 m from the radar, resulting in a standard deviation of $6.1^{\circ }$, which translates to an accuracy of 42 μm when converted into distance units. With this level of precision, changes in distance of a few millimeters can be accurately measured.

Additionally, by using pulse waves for transmission, unlike radar systems that use frequency modulation (known as frequency-modulated continuous-wave radar), there is no need to constantly transmit waves, facilitating extremely low power consumption. The power consumption of the radar is 81.3 mW at a transmission interval of 0.01 s.

## 3. Proposed Method to Identify Swallowing Using the Radar

### 3.1. Estimation Method of Change in Distance

**Figure 2:**
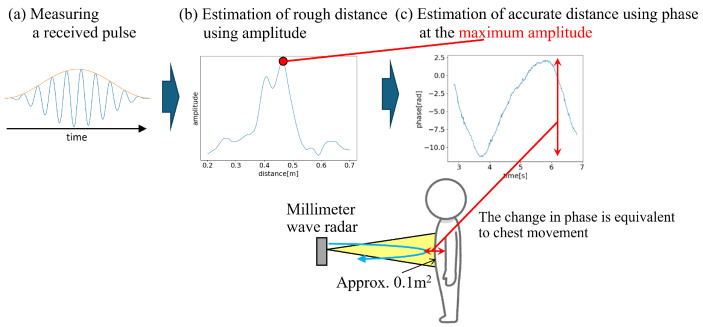
Proposed method to estimate the chest movement distance.

The measurement method is shown in Figure 2. To measure chest movements, data were first obtained from the sensor. The amplitude and phase information of the reflected signal were recorded for each transmission interval of the pulse wave, with a distance resolution of 1 mm. In this study, the transmission interval was set to 0.01 s.

The measurement range was set from 0.2 m to 0.7 m, and only the signals that returned within a certain delay time from the transmission of the pulse wave were used. Additionally, the radar was placed in front of the chest to ensure that the chest surface was within the measurement range. These restrictions on the reflection time and range help to avoid the problem of multipath reflections.

The chest movement distance was estimated using these steps. First, a certain amount of data was extracted from the overall measurement data, assuming that the participants maintained their posture during this interval. Subsequently, at each time point of the extracted data, the distance at which the amplitude value measured using the ToF principle reached its maximum was determined (Figure 2b). By performing this procedure for all extracted data, a rough estimate of the distance with a resolution of 1 mm based on the amplitude of the received data was obtained. To mitigate the influence of outliers caused by noise, the method used in this study involved estimating the distance to the object as the mode of the maximum amplitude values at each time point within the extracted interval.

Subsequently, the phase data at that distance were extracted. However, the phase is a real value ranging from [0,2π). If it crosses the boundary between 2π and 0, the value changes discontinuously. To address this issue, the differences between the data at the current and previous time points were compared. If this difference exceeded π, the phase was determined to have crossed the boundary, and the data were corrected by adding or subtracting 2π. The phase data obtained after this process are shown in Figure 2c. The changes in phase corresponded to the changes in distance, indicating that small changes in distance were reflected as significant changes in phase. Through these procedures, the measurement data obtained from the radar were acquired as data representing changes in the distance to the object.

Although a low-pass filter is commonly used for this type of measurement data, the amount of noise is small, as shown in Figure 2c. In addition, no outliers that needed to be removed were found. Therefore, we used raw data for the feature extraction process without any filtering or other processing.

### 3.2. Method for Identifying Swallowing and Respiring

The intervals of respiring, breath-holding, and swallowing were identified, based on data representing the chest movement distances obtained from the radar.

In this study, data were extracted from the entire measurement dataset at fixed intervals. Respiring, breath-holding, and swallowing were identified for each window. The window size was set to 4 s (400 frames) and was slid every 1 s (100 frames) for extraction. The extraction method and window size were based on a previous study by Kubo et al. [24]. The window size was verified by another experiment described in Section 4.5.3.

State-of-the-art techniques based on deep learning have been developed for identification. However, this study is the first to focus on the time interval including breathing temporarily pauses during swallowing. To confirm that traditional machine learning techniques often used for respiratory signals could achieve good performance, we two selected traditional machine learning methods: Support Vector Machine (SVM) [33] and Random Forest Classification (RFC) [34]. These traditional machine learning techniques are widely used in respiratory detection methods that use radar [23,24]. We anticipate that this method will be useful for identifying swallowing. These methods were applied using scikit-learn.

Traditional machine learning methods strongly depend on the extracted features. Therefore, these features were compared to confirm the dependency. In one case, features were designed using a limited number of features based on previous research [24]. In other cases, a large number of features are extracted considering the commonly used features for time-series data.

#### 3.2.1. Designed Features

Four parameters were calculated to represent the characteristics of the respiratory waveform and used as features for discrimination. The following parameters were used.

##### Range of Chest Movement

The range of chest movement can be used to distinguish between the presence and absence of respiration. During breath-holding, there is almost no anterior–posterior movement of the chest, resulting in small values, whereas during respiration, these values tend to be larger. Additionally, in this study, a measure that removed values close to the maximum and minimum, such as the interquartile range, was used. In general, the respiratory waveform resembles a sine wave, thereby maintaining a high interquartile range. Consequently, this is effective in identifying the presence or absence of respiration, while avoiding the influence of noise. Moreover, respiration temporarily pauses during swallowing, but the duration of this pause is minimal and thus has a minimal impact on the range of movement. Therefore, the range of chest movements is useful for identifying breath-holding intervals.

##### Periodicity of Respiration

**Figure 3:**
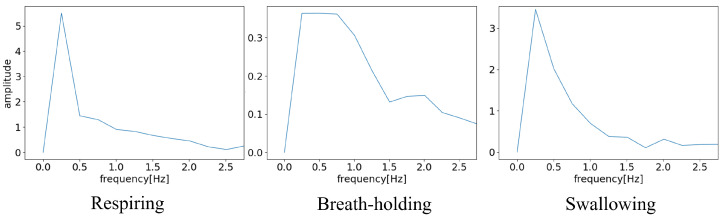
Results of FFT.

Parameters such as autocorrelation and FFT spectra can be used to reflect the periodicity of respiration. Respiration produces a signal resembling a sine wave with a cycle of approximately 4 s. Hence, calculating the autocorrelation or FFT tends to reveal peaks at specific values. In contrast, in cases of breath-holding or swallowing, where the periodicity is disrupted, the peaks become less pronounced. Therefore, these parameters may be useful for identifying respiratory intervals. In addition, as the DC component was not significant, it was removed by subtracting the mean signal within the window. Furthermore, because high-frequency signals carry no meaningful information, FFT was utilized to extract only low-frequency information corresponding to the respiratory cycle (Figure 3).

##### Histogram

**Figure 4:**
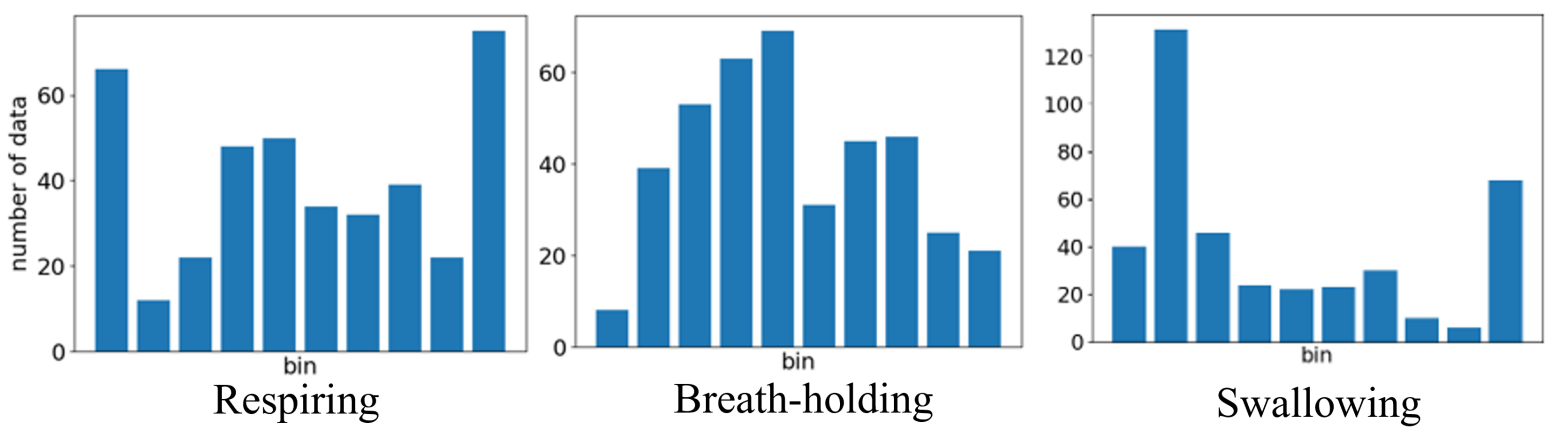
Results of histogram.

This feature was used to reflect the overall shape of the waveform. The data were discretized into a histogram by dividing the range from the maximum to the minimum value into ten equally spaced bins. Consequently, the widths and values of the intervals differ for each extracted dataset. Figure 4 is the calculated histogram in each condition. During breath-holding intervals, the histogram tends to exhibit a flat shape. In respiration intervals with short pauses between expiration and inspiration, and between inspiration and expiration, the histogram tends to form two peaks. During swallowing intervals, the histogram tended to have larger values during temporary respiratory pauses by swallowing. As the shape of the histogram varies according to the state, it is considered useful for detection.

For the range of chest movements, autocorrelation, FFT, and histogram, we extracted ten parameters, each as a feature. Thus, the dimensionality of the input used for training was 40.

#### 3.2.2. General Features

As a generally used method capable of computing numerous features, a Python library called tsfresh [35] was utilized. This library performs computations of numerous parameters that can serve as features, including statistical metrics such as mean and variance, making it suitable for time-series data such as the measurements in this study and sensor data.

The library extracted 783 features. However, some features had values that were either all zero or the same. These unnecessary features were removed using the feature-selection functionality of the library because they could potentially negatively impact on learning. In this study, the remaining 598 features were used for training.

## 4. Experiment for Performance Evaluation

### 4.1. Setting Up the Experimental Environment

To measure respiration using millimeter wave radar and evaluate the performance of respiring and swallowing interval detection, a measurement system was created to measure millimeter wave radar data with reference sensors (a band-type sensor and a camera) simultaneously.

**Figure 5:**
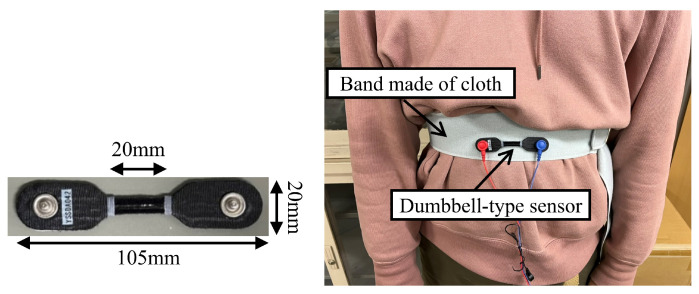
Band-type sensor.

A band-type sensor was used as a reference to confirm the feasibility of respiratory measurements using millimeter wave radar. A dumbbell-type sensor YSSDA042 (YAMAHA, Hamamatsu, Japan) [36] was used. The sensor utilizes carbon nanotubes as its material and detects the stretching and contraction of the sensor due to respiration through changes in resistance. In this experiment, the sensor was attached to a cloth band wrapped around the subject’s body (Figure 5). The band was wrapped around the lower ribs to ensure easy measurement of chest movements.

Furthermore, to extract the resistance changes in the sensor as a function of voltage, M5StickCPlus (M5Stack, Shenzhen China) was used for analysis. The sampling interval was set to 0.01 s to match that of the millimeter wave radar. Additionally, a Butterworth filter was employed as a low-pass filter for noise reduction. The cutoff frequency was set to 5 Hz, and the filter order was 4.

A camera was used to create ground truth data to identify swallowing intervals using millimeter wave radar. A C925e webcam (Logicool, Lausanne, Switzerland) was used to capture images of the throat every 0.1 s. Throat images were visually checked by confirming the laryngeal prominence. The swallowing interval was defined as the period from the onset of laryngeal elevation to the onset of descent, indicating the pharyngeal phase where the respiratory pause occurs. The method for creating ground truth data and defining the swallowing intervals followed the same approach as that used by Roldan-Vasco et al. [37].

The measurement system transmitted data to a PC via USB serial communication for data storage and time synchronization. The arrival time of the data on the PC was used to synchronize the sensors. Instructions for participants, such as swallowing or breath-holding, were provided through visual prompts displayed on the monitor. Controlling the timing of these instructions ensured uniform experimental conditions and provided reference information for verifying the timing of events.

### 4.2. Experimental Procedure

**Figure 6:**
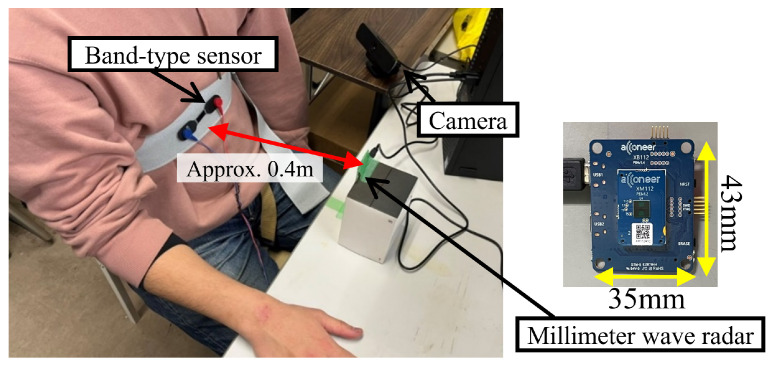
Experimental conditions.

This experiment was approved by the Ethics Committee of the Graduate School of Engineering, Osaka Metropolitan University (Approval Number: No22-01). The participants included 18 healthy males and 2 healthy females in their twenties. All participants underwent the RSST to confirm their normal swallowing function. The millimeter wave radar was installed at a height of 35 cm from the seat surface of a chair, simulating a typical household setup where the sensor was placed on a desk for measurement. The experimental setup is shown in Figure 6.

**Figure 7:**
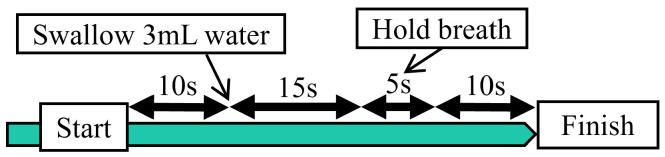
Flowchart of the experiment.

A flowchart outlining the procedure instructed to participants during the measurements is shown in Figure 7. The trial was conducted ten times for each participant. The amount of water to be swallowed was set to 3 mL, following the MWST protocol [13], which is commonly used as a screening test for swallowing function, similar to the RSST. Additionally, it is known that durations of temporary respiratory pauses during swallowing does not depend on the type and quantity of the substance [38], and their impact on the results is considered minor.

### 4.3. Confirmation of Chest Movement Measurement

**Figure 8:**
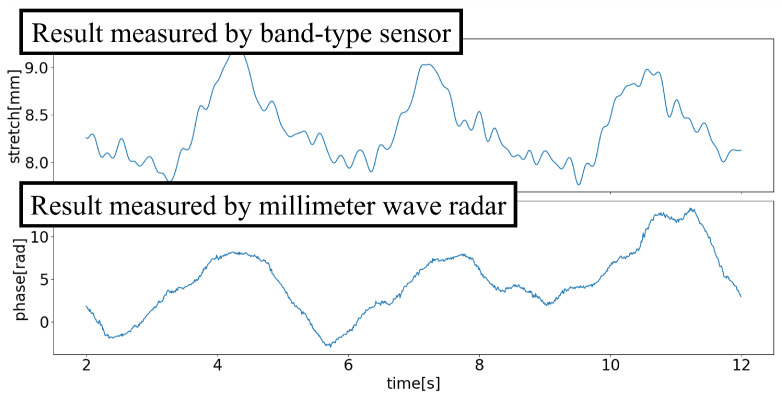
Result of the band-type sensor and the radar.

To validate the measurement of respiration with the millimeter wave radar, the radar data were compared with a reference band-type sensor, while the millimeter wave radar measured the anterior–posterior movement of the chest, the band-type sensor measured changes in chest circumference. Despite these differences, both the radar and band-type sensor data reflected the movement of the chest during respiration. Therefore, the waveforms measured by the millimeter wave radar corresponded with those measured by the band-type sensor. An example of chest movement measurement results obtained from the two sensors during respiration is shown in Figure 8.

**Figure 9:**
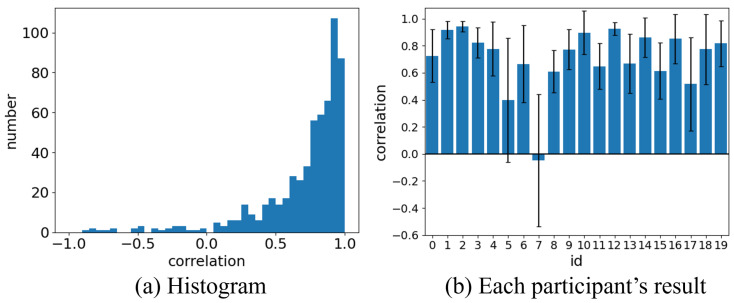
Correlation of respiratory waveforms between the band-type sensor and the radar.

Verification was conducted to confirm whether the waveforms matched, using cross-correlation as the metric. Cross-correlation is represented by real numbers ranging from −1 to 1, where higher values indicate a closer match between the two waveforms. Cross-correlation was used as a metric to assess the degree of similarity between the different datasets. Data for the evaluation were obtained from the experiments described earlier. Intervals containing only respiratory data were extracted, and each interval was set to a length of 5 s, which was sufficient to capture one full respiratory cycle. Since three intervals were used for each trial, 30 intervals were obtained per participant, resulting in 600 intervals for the entire dataset. The results of cross-correlation calculations are shown in Figure 9.

Overall, the mean value was 0.71 with a standard deviation of 0.32. The average value for the entire dataset exceeded 0.7, indicating a high correlation. The histograms of these correlations are shown in Figure 9a, where a peak exists in the range of 0.90 to 0.95. The mean correlation values for each participant, as shown in Figure 9b, are positive for all participants except one. These high correlations suggest that distance measurement of the chest using millimeter wave radar provides sufficient performance for respiratory cycle measurement. Variations in the mean and standard deviation values were observed among participants. These variations may be caused by individual differences in respiratory depth, sensor noise, and slight variations in millimeter wave radar data due to body movement. The main contributing factors to the lower performance were the relatively large noise in the band-type sensor for one participant (id:5 Figure 9b) and the comparatively significant body movement for one participant (id:17 Figure 9b).

Overall, these findings suggest that chest movements can be effectively measured using millimeter wave radar for most participants.

Only one participant (id:7 Figure 9b) showed a negative average correlation value of −0.02, indicating no correlation. A re-experiment was conducted on this subject, but the results did not change. Compared with the other participants, there were no apparent issues with the data from the band-type sensor for this participant. As there were no significant differences in physical characteristics such as height and weight, potential causes could include frequent minor body movements during the measurement or interference from other body parts, such as arms, within the measurement range of the millimeter wave radar. Therefore, the failure in measuring chest movement using the millimeter wave radar might not be due to the device itself or the measurement settings, but rather due to individual participant behavior. In this study, the data from this participant were excluded from further analysis and validation.

### 4.4. Confirmation of Respiratory Patterns before and after Swallowing

The ability to measure temporary respiratory pauses during swallowing and respiratory patterns before and after swallowing were confirmed using chest movement measurements by millimeter wave radar and throat images captured by a camera.

**Figure 10:**
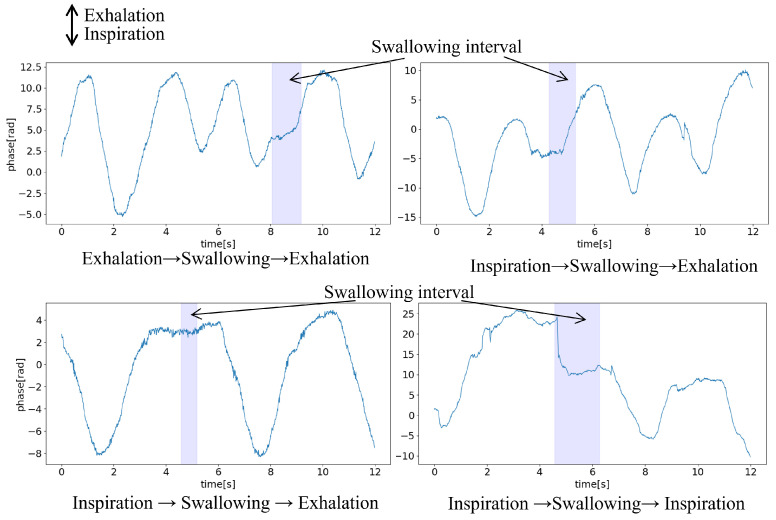
Respiratory pattern during swallowing.

Figure 10 illustrates an example of chest movement data during swallowing and its surrounding intervals. The y-axis represents the positive direction of expiration and negative direction of inspiration. The waveform captured by the millimeter wave radar can be used to observe the temporary respiratory pauses during swallowing by measuring the chest movement. Furthermore, the results demonstrated that the respiratory patterns before and after the swallowing interval, indicating whether expiration or inspiration can be robustly observed using millimeter wave radar.

For ten participants, excluding those for whom no correlation with the band-type sensor was observed, the visual classification of respiratory patterns before and after the swallowing interval was performed. The results are shown in Table 1. As each participant swallowed 10 times, and the total number of data points was 190.

For some data points, the respiratory patterns could not be discerned because of the absence of temporary respiratory pauses during swallowing. However, among the identifiable patterns, expiration before and after swallowing was the most common, whereas inspiration was the least common. All the participants in this study were young and healthy. Since it is common for healthy individuals to exhibit expiration after swallowing, this measurement method using radar was considered valid for confirming temporary respiratory pauses due to swallowing.

### 4.5. Automatic Identification of Respiration and Swallowing

#### 4.5.1. Creation of Dataset and Method of Identification

Automatic identification of the respiratory and swallowing intervals was performed using respiratory measurement data obtained from a millimeter wave radar. First, for each timestamp of the measurement data, the ground truth data indicating whether the subject was respiring, breath-holding, or swallowing interval were assigned by the researcher. Swallowing intervals were directly determined from the camera images, while breath-holding intervals were defined as the periods when the subjects were instructed to hold their breath. To account for the time between displaying the instructions on the monitor and the subject’s response, a delay of 0.5 s was applied to the breath-holding intervals.

The dataset is composed of the calculated features extracted from the collected data and their corresponding ground-truth labels. The ground truth labels were assigned based on the identified action at the midpoint of the window, except in cases where both the start and end of a swallowing interval were contained within the window, in which case it was labeled as swallowing. Consequently, a dataset comprising 6833 instances was created from the measured data of 19 participants, including 5272 instances of respiring data, 950 instances of breath-holding data, and 611 instances of swallowing data. The number of data points in this dataset was sufficiently large compared to other studies that applied machine learning techniques [23,24], making it possible to apply machine learning methods such as SVM and RFC.

#### 4.5.2. Consideration of Detection Methods

The dataset was highly imbalanced in each class, with the majority of instances belonging to the respiring class, threatening that this imbalance would decrease the classification performance. To address this issue, larger weights were assigned to minority classes during training, namely breath-holding and swallowing data. In addition, for the performance evaluation, the macro-averaged F1 score was used, a metric that equally emphasizes all classes, ensuring a balanced evaluation across classes. The hyperparameters were C and gamma (gamma applied only to the rbf kernel) for SVM. For RFC, the number and depth of decision trees, and the number of features used in each decision tree were utilized. Hyperparameter tuning was performed to maximize this metric.

Results of three-class classification of respiring, breath-holding, and swallowing are listed in Table 2. Owing to the extensive computation time, SVM with an RBF kernel was not applied to the general features extracted using tsfresh.

The results indicated that the designed features generally achieved a higher discriminative performance. This result indicated that the ranges of chest movements, periodicity, and waveform shapes introduced in Section 3.2.1 were instrumental in capturing the characteristics of the respiratory waveforms during respiring and swallowing. Although tsfresh employs similar techniques for feature computation, it is speculated that the high dimensionality of the features relative to the dataset size leads to overfitting and, consequently, inferior performance. For subsequent validation, only RFC with the designed features was used considering this result.

In addition, the respiratory cycle of each subject was calculated based on the results of the autocorrelation computation. As a result, the mean value was 3.80 s with a standard deviation of 0.93 s, while the respiratory cycle is generally considered to be approximately 4 s, it strongly depends on individual variations. In our experiment, the participants’ respiratory cycles ranged from as fast as 2.5 s to as slow as 5 s, representing an almost two-fold difference. Although it is plausible that this difference contributed to the decreased discriminative performance, the estimation of swallowing and respiring was still feasible. Therefore, our machine learning method can estimate every phase without individual differences in the respiratory cycles. Furthermore, the respiratory cycle of healthy individuals generally ranges from approximately 3.3 to 5 s [39]. Therefore, while some individuals may show a slight tendency towards tachypnea, the respiratory cycles of the subjects were within the appropriate range.

#### 4.5.3. Consideration of Window Width

**Figure 11:**
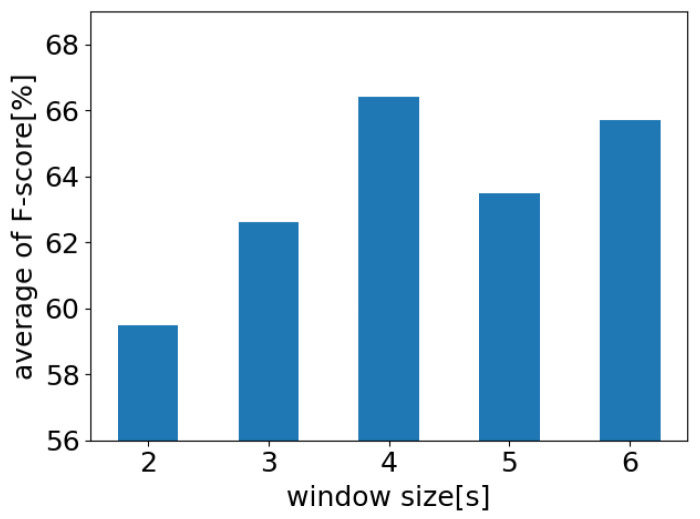
Relationship between window size and identification performance.

As described in Section 3.2, the window size extracted from the measurement data was set to 4 s based on the work of Kubo et al. [24]. However, in this study, the verification was conducted for other durations. The datasets were created by varying the window size from 2 to 6 s in increments of 1 s. For each duration, the experimental conditions and evaluation methods were the same as those used in the previous experiment. The results are shown in Figure 11.

The 4 s window size exhibited the best performance. However, when shortened to 2–3 s, a significant decrease of over 3% in the discriminative performance was observed. This decrease may be attributed to the absence of a complete respiratory cycle during this period, making it difficult to confirm the periodicity of respiration. In addition, setting the window size to 5–6 s, which was longer than the respiratory cycle, did not improve the performance. Consequently, the results suggested that a window size of 4 s, which is close to the length of one respiratory cycle, is appropriate for phase estimation.

#### 4.5.4. Consideration of Swallowing and Respiring Identification Performance

Confusion matrix for identification results of respiring (resp.), breath-holding (hold.), and swallowing (swal.) is presented in Table 3. The detection accuracy of the swallowing intervals appears to be inferior to that of the other methods, indicating difficulty in distinguishing between swallowing and respiration.

Binary classification of respiring and swallowing was performed by excluding the breath-holding data from the dataset. The results are summarized in Table 4. The macro-averaged F1 score was 64.7. Furthermore, for the detection of swallowing intervals, the precision was 31.3%, and the recall was 52.5%.

**Figure 12:**
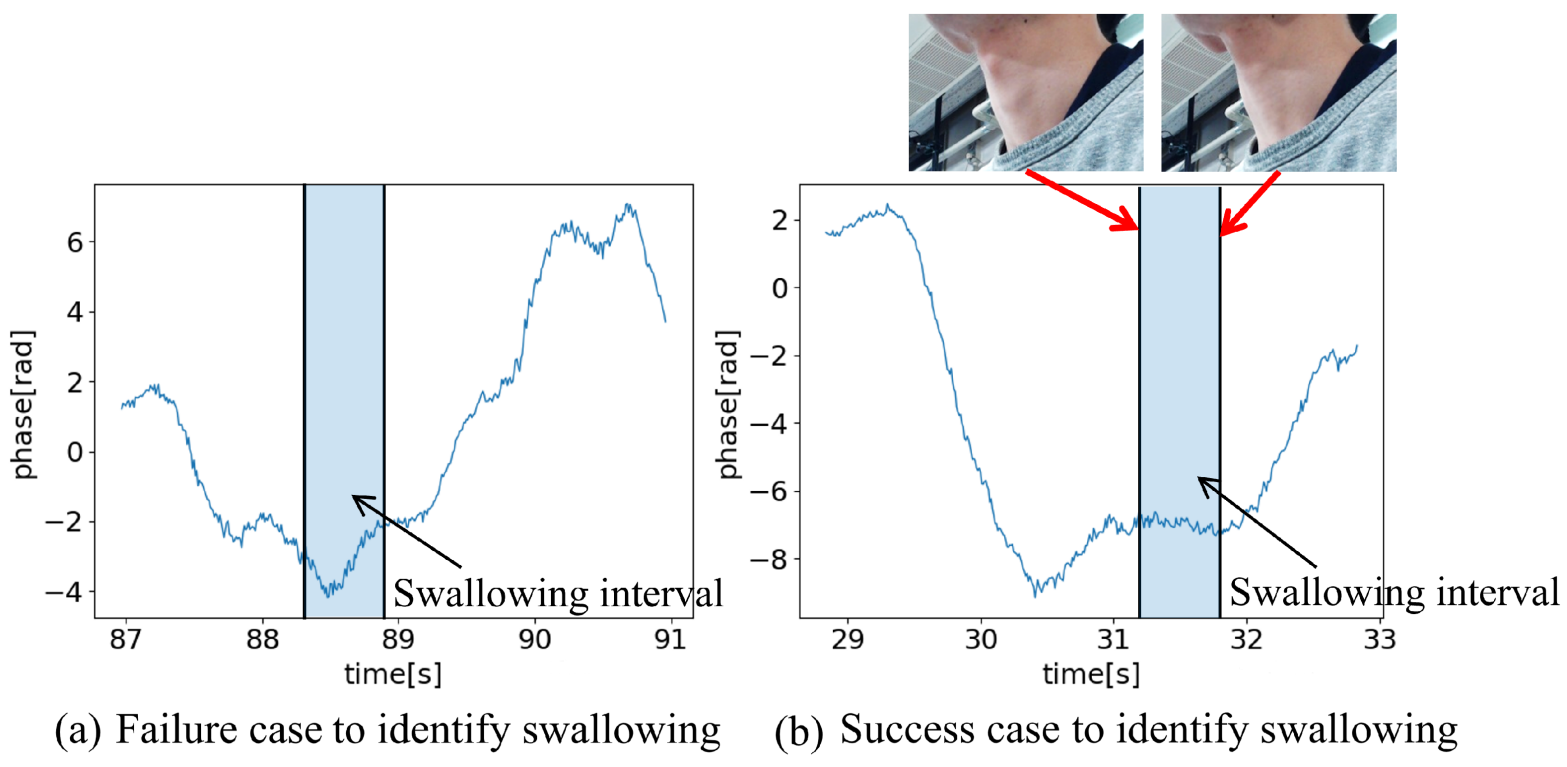
Example data of swallowing.

Figure 12 illustrates example data of swallowing. The interval from the beginning of the laryngeal elevation to the beginning of its descent is depicted in blue. As laryngeal elevation and temporary respiratory pause are coordinated, breathing temporarily stops during this period [15].

As an example of a misidentification, Figure 12a illustrates a case where swallowing data were incorrectly classified as respiring. During swallowing, respiratory pauses temporarily, leading to the absence of chest movements. Although pauses in chest movements can be detected using radar measurements, subtle body movements impede accurate identification, resulting in a decreased classification accuracy. However, it is evident that the respiratory waveform could be clearly measured in this case. By identifying the timing of swallowing using other methods, verifying the coordination between swallowing and respiration, which is considered to be related to swallowing function, is possible.

Conversely, for cases, such as those shown in Figure 12b, where the temporary pause in the chest movements during swallowing was clearly observed, and the method could distinguish between swallowing and respiring accurately. To improve the accuracy, methods capable of identifying swallowing intervals, even in the presence of minor body movements, are required.

**Figure 13:**
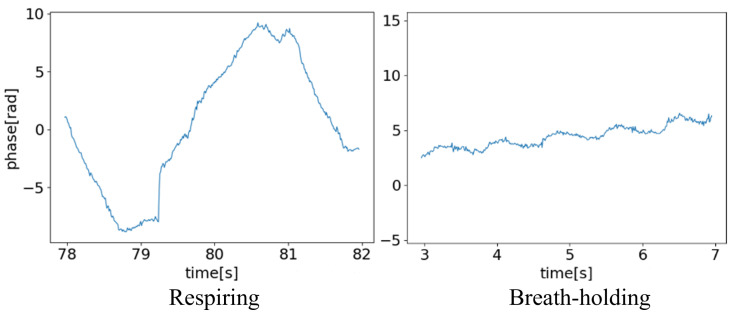
Example of respiring data.

On the other hand, a relatively high classification accuracy was achieved in distinguishing between respiring and breath-holding. By excluding swallowing data from the dataset, a binary classification between respiring and breath-holding was conducted. The results are summarized in Table 5. The macro-averaged F1 score is 88.5%. Furthermore, for the detection of breath-holding intervals, the precision was 80.1 These values are higher than those obtained for swallowing detection in all aspects. This indicates that detecting swallowing intervals is more challenging than detecting breath-holding intervals. As illustrated in Figure 13, breath-holding exhibited a noticeable decrease in chest movement compared with respiring. Although chest movements caused by breathing were minimal, they could be measured accurately.

In a study by Kubo et al. [24], breath-holding interval detection exhibited a precision of 64.1% and a recall of 54.3%. The results of our study demonstrate that the proposed method achieves high accuracy in interval detection. Furthermore, the results indicate that radar-based chest movement measurement techniques similar to those employed in microwave systems can be effectively achieved with millimeter wave radar. Moreover, the increase in distance resolution owing to the use of high-frequency millimeter waves likely contributed to the advantageous detection performance. However, methods for swallowing detection using contact sensors such as nasal cannulae or sEMG have already established a sufficient level of accuracy for reliable detection of swallowing. To achieve an accuracy comparable to that of methods using contact sensors, addressing issues specific to non-contact sensors, such as dealing with body movement, is necessary. In addition, if the body movement is so significant that breathing measurement becomes impossible, using another non-contact sensor to detect the body movement and repeat the measurement is feasible.

A limitation of this study is that all participants were young. However, as many individuals with dysphagia are elderly, the generalizability to such populations is limited. Differences in physique due to age disparities may affect measurement performance. Therefore, verifying whether the methods used in this study can also be applied to older adults is necessary.

Additionally, the number of participants in this experiment was small with only 20 participants. While it is valuable that respiratory measurements and swallowing interval detection can be performed with a certain level of accuracy, even in situations where there are significant individual differences, such as respiratory cycles, a larger amount of data is necessary to demonstrate the effectiveness of the method more clearly.

In this study, one subject’s data did not show any correlation with the reference data. Although the cause is currently unknown, if similar occurrences arise as more experimental data are collected, deeper investigation and validation will be necessary.

## 5. Conclusions

In this study, we propose a method for chest movement measurement using millimeter wave radar as an unconstrained and non-contact method for capturing physiological signals. The feasibility was demonstrated by measuring the respiratory waveforms from the acquired data and identifying temporary respiratory pauses that occur during swallowing and the respiratory patterns before and after these pauses. In terms of respiratory measurements, our method demonstrated a high correlation of 0.71 between the radar and the sensor used as a reference. Furthermore, the classifications of swallowing and respiring intervals using machine learning, resulting in an accuracy of 66.4%. In addition, a high accuracy of 88.5% was achieved in identifying the respiration. Potential for identification of swallowing intervals based on temporary respiratory pauses during swallowing has been demonstrated; although, there is room for improvement in its accuracy. These results suggest the potential use of the millimeter wave radar for monitoring the daily activities of older adults.

For future research, the integration of other unconstrained sensors to improve classification accuracy is planned. In addition, the implementation of algorithms for data collection and estimation using multiple sensors is required. Furthermore, obtaining data from individuals with dysphagia in the future to determine whether this method can be used to evaluate swallowing function will be necessary.

Furthermore, with increased accuracy in measurement and identification, it becomes possible to confirm the coordinated relationship between swallowing and respiration, which is believed to be related to swallowing function [16,17]. Once individuals whose coordination is disrupted are detected early without constraint during daily activities, interventions such as encouraging exercises to improve swallowing ability can be used to recover swallowing function, which will lead to pneumonia prevention.

## Figures and Tables

**Table 1:** Respiratory patterns before and after swallowing interval.

Respiratory Pattern	Number
Exhalation → Swallowing → Exhalation	85
Inspiration → Swallowing → Exhalation	46
Exhalation → Swallowing → Inspiration	27
Inspiration → Swallowing → Inspiration	10
Indistinguishable	22

**Table 2:** Comparison of identification performance.

	Designed Features	General Features
SVM (rbf)	65.3%	——
SVM (linear)	62.4%	62.0%
RFC	66.3%	63.5%

**Table 3:** Results of 3-class classification.

		Pred.		
		resp.	hold.	swal.
	resp.	4543	171	558
Ans.	hold.	79	747	124
	swal.	283	72	256

**Table 4:** Results of classification between respiring and swallowing.

		Pred.	
		resp.	swal.
Ans.	resp.	4569	703
	swal.	290	321

**Table 5:** Results of classification between respiring and breath-holding.

		Pred.	
		resp.	hold.
Ans.	resp.	5081	191
	hold.	182	768

## Data Availability

There are no open data related to this research because informed consent did not include data opening.
